# Amick

**Published:** 1893-09

**Authors:** 


### EDITORIAL DEPARTMENT.

F. E. DANIEL, M. D., Editor.
S. E. HUDSON, M. D., Managing Editor.
A. J. SMITH. M. D., Galveston, Associate Editor.

EDITORIAL STAFF:

PROF. J. E. THOMPSON, M. D., Texas Medical College, Galveston; Surgery.
PROF. WM. KEILLER, M. D., Texas Medical College, Galveston; Obstetrics and Gynecology.
PROF. DAVID CERNA, M. D., Texas Medical College, Galveston; Therapeutics.
PROF. A. J. SMITH, M. D., Texas Medical College, Galveston; Medicine.
DR. ISADORE DYER, Tulane University; Dermatology.
DR. R. H. L. BIBB, Saltillo, Mexico; Foreign Correspondent.
DR. ROBT. MORRIS, Charity Hospital, N. O.; Clinical Reports

———o———

The JOURNAL is the official organ of the Austin District Medical Society, the West Texas District Medical Society and the Galveston County Medical Society.

AMICK.—When a man turns quack he turns quack all over. New converts shout loudest. Amick is trying to leave not a peg on which to hang a doubt that he is an out and out quack. The JOURNAL was the very first to denounce his claims for his so-called "chemical cure for consumption" as rank quackery, and his method of geting it published in the *Lancet Clinic* as in keeping with the first principles of the born quack. It will be remembered that W. R. Amick was a reputable physician in Cincinnati, and was honored with a professorship in the Cincinnati College of Physicians and Surgeons. As such his writings for the *Lancet Clinic* were always acceptable, and his office being near by, the editors of the *Lancet Clinic* let him read proof often. He took advantage of this confidence to publish the first claims to having discovered an infallible *cure* for consumption, and brought great odium on that journal. A prominent physician in Cincinnati wrote us that our editorial, denouncing the publication and the whole thing, created a sensation in medical circles in that city, and that Amick would surely be turned out of the college. He was; and then it was, that like Rickard III, who said "since I cannot be a hero I will be a villain," he flew to the sheltering arms of quackdom, and determined to out quack the last mother's son of them.

His "chemical cure" was and is extensively advertised in the newspapers. At first the *medical journals* would not touch it, but the *Maryland Medical Journal* we believe, made the first break,—was tempted by the money there was in it,—and has been followed by several others, notably, recently, by the *St. Louis Weekly Medical Review*, owned and published by Chambers & Co., the prime movers now in a step to organize medical publishers for mutual benefit and protection,—as *we* thought, against just such imposition as this outrageously quack advertisement! These journals are thus helping Amick to foist his worthless stuff not only upon the people but upon the very profession which kicked him out as being unworthy of recognition; for he has the unparalleled assurance to announce that his medicines and fixtures,—his "chemical cure,"—his "constitutional drops" to put the constitution in order, his powders to "allay irritation,' his pills to "supply the lacking constituents," his *inhaler* (patented) to "soothe the bronchial irritation,"—the lay-out,—can only be had *through reputable physicians*, that he will send "any *reputable physician* enough chemical cure for a trial,"—as if "any reputable physician" in knowledge of the facts would touch him or his "cure" with a forty-foot pole. But there is where the trouble comes in. Many really respectable physicians have been *deceived* by his shrewd management and the co-operation of the *medical press* in part, and by newspaper reports,—into *ordering* his stuff; and it is for the purpose of warning our readers of these tricks this is written. His latest move is to manufacture reports of cures or procure them from the pliable, and have them inserted in newspapers all over the country as *Associated Press dispatches!* One appeared here in Austin, in the *Daily Statesman*, a report of some wonderful cure by some wonderful doctor, under the guise of a dispatch by the Associated Press, and an old editor long connected with the press of this city, informed us that the editor of the Statesman admitted to him that it was a *paid advertisement*, extra charge for the position given it in the telegraph columns. Comment would be superfluous.

